# The Epidemic of Non-Suicidal Self-Harm in Adolescents and Young Adults in the Kyrgyz Republic

**DOI:** 10.1192/j.eurpsy.2024.1645

**Published:** 2024-08-27

**Authors:** E. Molchanova

**Affiliations:** Psychology, American University of Central Asia, Bishkek, Kyrgyzstan

## Abstract

**Introduction:**

During the last two years, many young people and adolescents in the Kyrgyz Republic started to visit specialists due to Non-suicidal self-harm. A significant rise in the amount of such cases allowed specialists from the Republican Center of Psychiatry in Bishkek to think about an “epidemic “of self-harming behavior.  Non-suicidal self-harm (NSSH) is defined as repeated, deliberate, direct injury to the body without suicidal intent that is not socially acceptable (Nixon et al., 2002) to reduce psychological discomfort in the absence of a conscious intention to take one’s life (ICD-10 codes X60-X84, and ICD-11 codes PB80-PD3Z).

**Objectives:**

To determine the causes of NSSH among adolescents and young adults who approached specialists in mental health sphere at Institute of Behavioral Health at the American University of Central Asia.

**Methods:**

Over two years, forty-five adolescents and young adults under twenty-five who had committed self-harm visited specialists from the Behavioral Health Institute at the American University of Central Asia.

All the patients received either dialectical behavioral treatment or cognitive processing treatment, medication (paroxetine) was used in three cases.

**Results:**

Thirty patients were girls under twenty-one, and fifteen were boys and young male adults. The overwhelming majority (40 people) had self-inflicted cuts, two had imposed burns with matches and cigarettes, and one had used self-suffocation without a bond. Reasons for self-harm were the following: releasing internal tension and anxiety, getting some rest from intrusive thoughts, relieving the inner pain, and a desire to “feel as a whole person.” All the patients underlined that they did not want to attract attention from their family members; moreover, they tried to hide the consequences of self-harm.

Teens and young adults (twelve patients) from Kyrgyz traditional families visited a consultant or psychiatrist after a long drive through conventional or religious healers. All of the patients knew that they were addicted to self-harm, wanted to stop a problematic behavior, and could not stop it on their own. Ten patients have been diagnosed with borderline personality disorder. Two of them also had eating disorders. Five patients had PTSD, and five had social phobia. The others had recognizable anxiety symptoms.

**Conclusions:**

The enormous rise of non-suicidal self-harm is a phenomenon that needs further research. Those cases often resist treatment due to the “addictive” component in the pathogenesis.

**Disclosure of Interest:**

None Declared

